# Supplementary material for: Cloud BioLinux: pre-configured and on-demand bioinformatics computing for the genomics community
Source: BMC Bioinformatics. 2012 Mar 19;13:42. doi: 10.1186/1471-2105-13-42 (PMC3372431; doi:10.1186/1471-2105-13-42)
Supplement: Additional file 1 — Supplementary 1 Cloud BioLinux software documentation in the form of a mini, self-contained website. Users need to download and uncompress the .zip file, and open through a web browser the "index.html" file available on the main directory. (ZIP 1823 kb). [file 1471-2105-13-42-S1.ZIP › Cloud-BioLinux-Package-Documentation/list_cat.html]

Bio-Linux Software Documentation Pages

Back to search form

### Browse by Category

|  |  |  |  |  |  |  |  |  |
| --- | --- | --- | --- | --- | --- | --- | --- | --- |
| Acd; acdvalid acdtrace acdtable acdpretty acdlog acdc | Alignment; FastTree dialign \|; Alignment > Consensus; cons consambig gap4 megamerger merger spin; Alignment > Differences; act diffseq; Alignment > Dot\_plots; dotmatcher dotpath dotter dottup polydot; Alignment > Editing; clcsequenceviewer jalview squint; Alignment > Global; est2genome fasta ggsearch glsearch needle needleall stretcher swat; Alignment > Graphical; arb bl2seq bwa cross\_match dba fasta fastf fastm fasts fastx fasty flalign glsearch lalign lfasta matcher plalign plfasta prenuc prfx prss psw pswdb seqmatchall sibsim4 sim4 ssearch supermatcher swat tfastf tfastm tfasts tfastx tfasty water wordfinder wordmatch; Alignment > Multiple; annotate clustalw clustalx combineMUMs dialign edialign emma exact-tandems gaps hmmalign infoalign jalview lalign2list mapview mgaps mocca mummer mummerplot muscle nucmer nucmer2xfig plotcon postnuc postpro prank prepro prettyplot PROmer repeat-match run-mummer1 run-mummer3 seaview show-aligns show-coords show-tiling showalign squint t-coffee tranalign yamap; Alignment > Profiles; pfmake pfsearch pftools psa2msa ptof ptoh; Alignment > Statistics; alistat pfscale prfx prss weight | Clustering; \|; Clustering > Graph; clmconf clmdist clmimac clminfo clmmeet clmresidue mcl mcx mcxconvert mcxmap mcxsubs; Clustering > Sequences; assembly-conversion-tools blastclust cd-hit clobb gap4 gcphrap phrap qiime uclust | Databases; omssa big-blast \|; Databases > Indexing; arb cdbfasta formatdb formatrpsdb hmmindex makeblastdb makembindex sindex; Databases > Post\_search\_graphical; mview; Databases > Post\_search\_processing; mspcrunch mview prfx prss; Databases > Retrieval; afetch arb blastdbcmd cdbyank fastacmd hmmfetch maxdLoad2 sfetch; Databases > Searching; arb big-blast blast+ blast2 blastall blastcl3 blastn blastp blastpgp blastx fasta fastf fastm fasts fastx fasty ggsearch glsearch hmmpfam hmmpfam-pvm hmmsearch hmmsearch-pvm impala maxdLoad2 megablast netblast omssa pfscan pfsearch psiblast rpsblast rpsblast+ rpstblastn seedtop ssearch taxblast tblastn tblastx tfastf tfastm tfasts tfastx tfasty; Databases > Submission\_tools; sequin | Display; cytoscape showpep cn3d textsearch sixpack showseq showdb seealso remap prettyseq pepwheel pepnet lindna cirdna abiview trev \|; Display > Alignments; boxshade jprofilegrid; Display > Annotation; act artemis gff2ps showfeat; Display > Dotplots; dotter lalign lav2ps lav2svg; Display > Sequence\_traces; consed gap4 trev; Display > Structure; cn3d rasmol | Edit; splitsource sizeseq seqretsetall nthseqset notab nospace nohtml aligncopypair yank vectorstrip union trimspace trimseq trimest splitter skipseq skipredundant seqrettype seqretsplit seqretset seqretall seqret revseq pasteseq origunion origsplitter nthseq notseq noreturn newseq maskseq maskfeat maskambigprot maskambignuc makeprotseq makenucseq listor featreport featcopy extractseq extractfeat extractalign entret descseq degapseq cutseq codcopy biosed aligncopy | Enzyme\_kinetics; findkm | Feature\_tables; twofeat coderet | Hmm; sreformat sindex shuffle sfetch hmmsearch-pvm hmmsearch hmmpfam-pvm hmmpfam hmmindex hmmfetch hmmemit hmmconvert hmmcalibrate-pvm hmmcalibrate hmmbuild hmmalign |
 Information; inforesidue infobase wossname whichdb tfm infoseq | Menus | Microarray; bioconductor \|; Microarray > Analysis; bioconductor genespring maxdLoad2 maxdview; Microarray > Annotation; bioconductor; Microarray > Graphical\_views; bioconductor genespring maxdview; Microarray > Primers; OligoArray | Nucleic; \|; Nucleic > 2d\_structure; arb einverted garnier helixturnhelix hmoment newcoils pepcoil pepnet pepwheel tmap; Nucleic > Annotation; act artemis partigene qiime rdp\_classifier; Nucleic > Assembly; bwa cap3 consed gcphrap mira partigene phrap ssake velvet; Nucleic > Base\_calling; phred pregap4; Nucleic > Codon\_usage; cai chips codcmp codon-usage cusp syco; Nucleic > Composition; backtranambig backtranseq banana btwisted chaos charge checktrans complex compseq compseq dan density emowse freak freak get-len giep iep isochore mwcontam mwfilter octanol pepinfo pepstats pepwindowall sirna wordcount wordcount; Nucleic > Cpg\_islands; cpgplot cpgreport geecee newcpgreport newcpgseek; Nucleic > Est; cd-hit-est estscan estwise estwisedb genewise genewisedb partigene prot4est sibsim4 sim4 trace2dbest; Nucleic > Gene\_finding; act anomaly artemis build-icm check codon-usage compare-lists estscan estwise estwisedb extract generate genewise genewisedb get-putative getorf glimmer2 long-orfs marscan plotorf prot4est run-glimmer2 showorf sibsim4 sim4 sixpack syco tcode wobble; Nucleic > Graphical; act artemis; Nucleic > Masking; dust mdust; Nucleic > Motifs; antigenic digest dreg epestfind fuzznuc fuzzpro fuzztran fuzztran glam2 helixturnhelix marscan ocount oddcomp patmatdb patmatmotifs pepcoil sigcleave; Nucleic > Mutation; msbar msbar pregap4 shuffleseq shuffleseq; Nucleic > Nextgen; bwa caftools consed denoiser maq mira mira-3rd-party qiime roche2gap samtools ssake tqs velvet; Nucleic > Primers; eprimer3 OligoArray prima primer3 primers primersearch stssearch; Nucleic > Profiles; jprofilegrid profit profit prophecy prophecy prophet prophet; Nucleic > Repeats; cross\_match dust dustmasker einverted equicktandem etandem mdust palindrome windowmasker; Nucleic > Restriction; recoder redata remap restdist restml restover restrict showseq silent; Nucleic > Transcription; jaspscan tfscan; Nucleic > Translation; 6ft backtranambig backtranseq coderet plotorf prettyseq remap showorf showseq sixpack tfastx tfasty transeq | Phylogeny; jMOTU phylip FastTree mothur evolver \|; Phylogeny > Bayesian; migrate mrbayes mrbayes-multi qiime; Phylogeny > Clustering; dotur qiime; Phylogeny > Consensus; consense; Phylogeny > Continuous\_characters; contml contrast; Phylogeny > Discrete\_characters; clique dollop dolpenny factor mix penny; Phylogeny > Distance\_matrix; arb fitch kitsch mothur neighbor; Phylogeny > Gene\_frequencies; gendist; Phylogeny > Maximum\_likelihood; arb baseml basemlg codeml fastdnaml mcmctree pamp Phyml proml PROmlk restml tree-puzzle yn00; Phylogeny > Maximum\_parsimony; dnamove pars; Phylogeny > Misc; bioperl extract\_from\_pdb mesquite pedro qiime seqsplit; Phylogeny > Molecular\_sequence; clustalw clustalx distmat dnacomp dnadist dnainvar dnaml dnamlk dnapars dnapenny protdist protpars qiime seqboot; Phylogeny > Tree\_drawing; ape arb atv dendroscope njplot qiime retree splitstree treedist treeview xsplits | Pipelines; Taverna run-mummer3 nucmer \|; Pipelines > 16S processing; qiime; Pipelines > Sequence\_assembly; gap4 partigene stars; Pipelines > Sequence\_processing; nucmer pregap4 run-mummer3 stars trace2dbest; Pipelines > Sequence\_typing; stars; Pipelines > Workflows; Taverna | Protein; omssa \|; Protein > 2d\_structure; arb einverted garnier helixturnhelix hmoment newcoils pepcoil pepnet pepwheel tmap; Protein > 3d\_structure; garlic psiphi rasmol; Protein > Composition; backtranambig backtranseq banana btwisted chaos charge checktrans complex compseq compseq dan density emowse freak freak get-len giep iep isochore mwcontam mwfilter octanol pepinfo pepstats pepwindowall sirna wordcount wordcount; Protein > Dynamics; gromacs; Protein > Mapping; peptidemapper; Protein > Masking; dustmasker segmasker windowmasker; Protein > Motifs; antigenic digest dreg epestfind fuzznuc fuzzpro fuzztran fuzztran glam2 helixturnhelix marscan ocount oddcomp patmatdb patmatmotifs pepcoil sigcleave; Protein > Mutation; msbar msbar pregap4 shuffleseq shuffleseq; Protein > Profiles; jprofilegrid profit profit prophecy prophecy prophet prophet | Statistics; mothur \|; Statistics > biodiversity; BiodiversityR mothur; Statistics > General; chi2 R Rcmdr; Statistics > Microarray; bioconductor; Statistics > Other\_sequence; fasta prfx prss seqstat; Statistics > qtl; BTmapqtl Emap Eqtl JZmapqtl LRmapqtl MImapqtl MultiRegress Preplot Prune Qstats QTLcart QTL\_Cartographer Rcross Rmap Rqtl SRmapqtl Zmapqtl | Test; testplot seqretsingle seqretallfeat seqinfo patmattest intconv histogramtest demotable demostringnew demostring demolist demofeatures demoalign corbatest ajtest ajfeatest ajbad | Utils; biojava extract\_from\_pdb bioperl \|; Utils > Assembly reformatting; assembly-conversion-tools caf2gap caftools gap2caf mira-3rd-party; Utils > Database\_creation; aaindexextract cutgextract jaspextract printsextract prosextract rebaseextract tfextract; Utils > Database\_indexing; dbiblast dbifasta dbiflat dbigcg dbxfasta dbxflat dbxgcg dbxstat; Utils > Misc; cytoscape dendroscope dolmove drawgram drawtree embossdata embossversion entrails Lamarc mesquite move njplot; Utils > Sequence\_cleaning; chimeraslayer denoiser lucy qiime; Utils > Sequence\_info; extract\_from\_pdb FastQC seqstat; Utils > Sequence\_reformatting; 2ft clustalw clustalx copymat extract\_from\_pdb gtop hmmconvert htop makemat pfmake psa2msa ptof ptoh readseq sfetch sreformat; Utils > Software\_development; bioconductor biojava bioperl bioruby |
